# CD24 promoted cancer cell angiogenesis via Hsp90-mediated STAT3/VEGF signaling pathway in colorectal cancer

**DOI:** 10.18632/oncotarget.10971

**Published:** 2016-08-01

**Authors:** Xinying Wang, Yu Zhang, Yingying Zhao, Yanling Liang, Cheng Xiang, Huanyu Zhou, Hui Zhang, Qiang Zhang, Haitao Qing, Bo Jiang, Huabao Xiong, Liang Peng

**Affiliations:** ^1^ Department of Gastroenterology, Nanfang Hospital, Southern Medical University, Guangzhou 510515, China; ^2^ Guangdong Provincial Key Laboratory of Gastroenterology, Guangzhou 510515, China; ^3^ Department of Gastroenterology, The First People's Hospital of Yunnan Province, Kunming 65003, China; ^4^ Department of Ultrasound Imaging, 306 Hospital of PLA, Beijing 100101, China; ^5^ Institute of Immunology and Molecular Medicine, Jining Medical College, Jining 272067, China; ^6^ Department of Medicine, Immunology Institute, Icahn School of Medicine at Mount Sinai, New York 10029, NY, USA

**Keywords:** CD24, Hsp90, colorectal cancer, angiogenesis, VEGF

## Abstract

CD24 is involved in tumor progression of various cancers, but the effects of CD24 on tumor angiogenesis in colorectal cancer are still unknown. We aimed to investigate the underlying mechanism and role of CD24 on colorectal cancer (CRC) angiogenesis. Our data showed that the microvessal density (MVD) was related to the expression of CD24 in primary and metastasis CRC. Silencing of CD24 could dramatically decrease human umbilical vein endothelial cell (HUVEC) migration, invasion and tubule formation, but trivially affected cell proliferation. We also mechanically showed that silencing CD24 could downregulate the expression of VEGF via inhibiting the phosphorylation and translocation of STAT3. Moreover, Hsp90 was identified as the down-interaction protein of CD24 with co-immunoprecipitation assay and systematic mass spectrometry. Immunofluorescence results showed Hsp90 partly co-localized with CD24 in CRC cell membrane and there was a positive correlation between CD24 and Hsp90 expression in CRC tissues. We gradually evidenced that Hsp90 modulated the stability and degradation of CD24 in a proteasome-depended manner, and transferred the signal transmission from CD24 to STAT3. 17-AAG, a specific Hsp90, could abrogate the CD24 induce- HUVEC migration, invasion and tubule formation *in vitro* and *in vivo*. Collectively, our results suggested that CD24 induced CRC angiogenesis in Hsp90-dependent manner and activated STAT3-mediated transcription of VEGF. We provided a new insight into the regulation mechanism of tumor angiogenesis by exploring the role of CD24 in angiogenesis.

## INTRODUCTION

Human CD24 is a short mucin-like cell surface protein consisting of a small protein core linked to cytoplasm membrane raft domains through a glycosyl-phosphatidylinositol (GPI) anchor [1–3]. Many studies have demonstrated that CD24 was overexpressed in most cancer cells and was closely related to the cancer cell proliferation, invasion and metastasis. Furthermore, CD24 also was regarded as a poor prognosis marker in various cancer patients, such as breast cancer and colorectal cancer(CRC) [4]. This evidence suggested CD24 was an important oncogene in the colon tract. As we all known, angiogenesis is a pivotal event in tumor progression and metastasis. The “angiogenic switch”, by which tumors acquire the ability to grow exponentially and disseminate beyond their primary site is a fundamental component in our knowledge of cancer [5]. One study showed that the loss of CD24 may have a deleterious effect on angiogenesis occurring in the second stage of retinopathy of prematurity (ROP) development [6]. Salnikov AV *et al* found that the silencing CD24 with special mono-antibody inhibited the tumor cell proliferation and angiogenesis *in vivo* and *in vitro* [7]. Even so, the role of CD24 in angiogenesis in CRC is still ambiguous and needs to be further addressed. We hypothesized that CD24 might play a key role in CRC angiogenesis.

Previous data have confirmed that Vascular Endothelial Growth Factor (VEGF) acted as an important regulator in cell proliferation, and metastasis in many types of tumors [8, 9]. VEGF binding to VEGF receptor (VEGFR) induced the angiogenesis. Furthermore, blocking the VEGF signaling pathway inhibited tumor angiogenesis, development and metastasis [10, 11]. The drug targeting the VEGF or VEGFR had been applied to the clinical trial and showed an impressive positive effect on the cancer patients. This is demonstrated by the existence of bevacizumab, a humanized monoclonal antibody against VEGF. Bevacizumab combined with chemotherapy had led to prolonged survival in patients with metastatic CRC. Therefore, the reduction of VEGF expression or inhibition of VEGF-mediated signaling pathway in endothelial cells was an important strategy for the restriction of tumor angiogenesis [12–15]. G Niu *et al.* confirmed that STAT3 was directly related to VEGF induction in tumors, which suggested that targeting STAT3 for therapeutic intervention in cancer might disrupt angiogenesis induced by multiple tyrosine kinase [16], and STAT3 was an important transcription factors activating VEGF by binding to its promoter [16, 17]. Cao and his colleagues reported that CD24 up-regulation was associated with VEGF-A expression [18]. Though, accumulated evidences suggested that CD24 was associated with carcinogenesis and cancer metastasis, it was still unclear whether CD24 could change angiogenesis resulting tumor metastasis in CRC.

In the present study, we evaluated the potential role of CD24 on tumor angiogenesis in CRC and the underlying molecular mechanisms. Our results demonstrated that CD24 recruited and interacted with Hsp90 at lipid rafts, in turn increased the STAT3 activity and transcriptionally regulated the VEGF expression. Moreover, Hsp90 also modulated the stability and degradation of CD24 in a proteasome-depended manner. The treatment with specific inhibitor against Hsp90 suppressed CD24-induced CRC angiogenesis and VEGF production. This depended on the STAT3-mediated transcription of VEGF. Our study enhanced the understanding of the biologic role of CD24 in CRC migration and invasion, thus providing with helpful therapeutic strategies for CRC.

## RESULTS

### CD24 was associated with the angiogenesis in colorectal cancer

**Figure 1:**
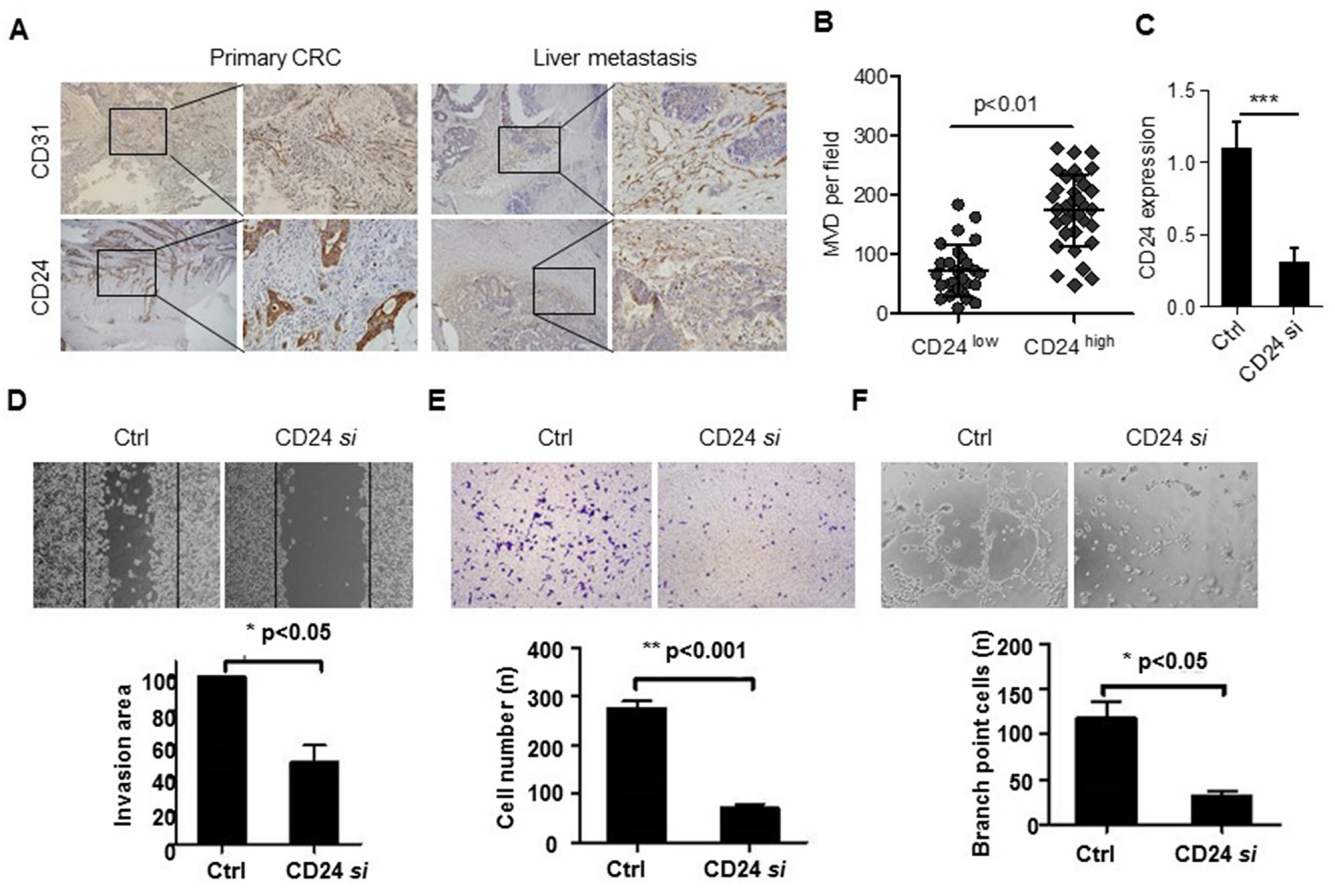
CD24 was related to the angiogenic properties of endothelial cells **A.** CD24 and CD31 were detected by IHC staining in serials specimens of primary CRC and liver metastatic tissues (n=7). **B.** The correlation of CD24 expression and MVD was analyzed by Spearman R (n=7). **C.** CD24 expression was analyzed by qPCR with the treatment of control (Ctrl) or CD24 siRNA (CD24 si). **D.** The conditioned Medium (CM) was obtained from HT29 cells which were respectively transfected with control siRNA (Ctrl) or CD24 siRNA (CD24si). The HUVEC migration was observed by wound-healing assay in the presence of CM. HUVECs were plated, scratched, and incubated with CM. Dotted lines, area occupied by the initial scraping. *, *p*<0.05. **E.** The HUVECs invasion was detected by Transwell assay in the presence of CM. Cells (2 x10^4^) were placed into the top chamber and CM was added to the bottom chamber. HUVECs were fixed, stained and counted under the microscope after 24h. **, *p*<0.001. **F.** The HUVECs tubule formation was analyzed in the presence of CM. Cells were seeded on top of ECM-matrix for 24h and tubular structures were counted using an inverted light microscope. ***, *p*<0.05. Original magnification: 100×.

Our previous study confirmed that CD24 was involved in the metastasis of colorectal cancer through MAPK signal pathway. Cell migration and invasion are two key steps for endothelial cells to form new blood vessels during angiogenesis processes. Our results showed that CD24 expression was closely related to MVD in primary colorectal cancer tissue and liver metastasis tissue (Figure 1A, 1B). *In vitro*, we performed wound healing assay, Boyden chamber migration and invasion assays to determine the effects of CD24 on HUVECs migration and invasion (*p*<0.05). As shown in Figure 1C, CD24 *si*RNA decreased the migration of HUVECs. To investigate the role of CD24 in HUVEC invasiveness, HT29 cells (a cell line with high expression of CD24, Supplementary Figure S1). invasion activity were tested after transfected with the control or CD24 *si*RNA. The percentage of cells that migrated through the filters from different groups was shown in Figure 1D and the invasion cells decreased significantly after transfected CD24 *si*RNA (*p*<0.001). Endothelial cells can spontaneously form a three-dimensional tubular capillary-like network in Matrigel culture *in vitro*. To examine the effects of CD24 on HUVECs tubule formation, we performed the tubule formation assayswith or without CD24 *si*RNA treatment. As shown in Figure 1E, silencing CD24 significantly inhibited HUVEC tubule formation (*p*<0.05). Our results showed that CD24 affected HUVEC migration, invasion, tubule formation and angiogenesis *in vitro*.

### CD24 promoted VEGF expression depending on STAT3 signaling pathway

**Figure 2:**
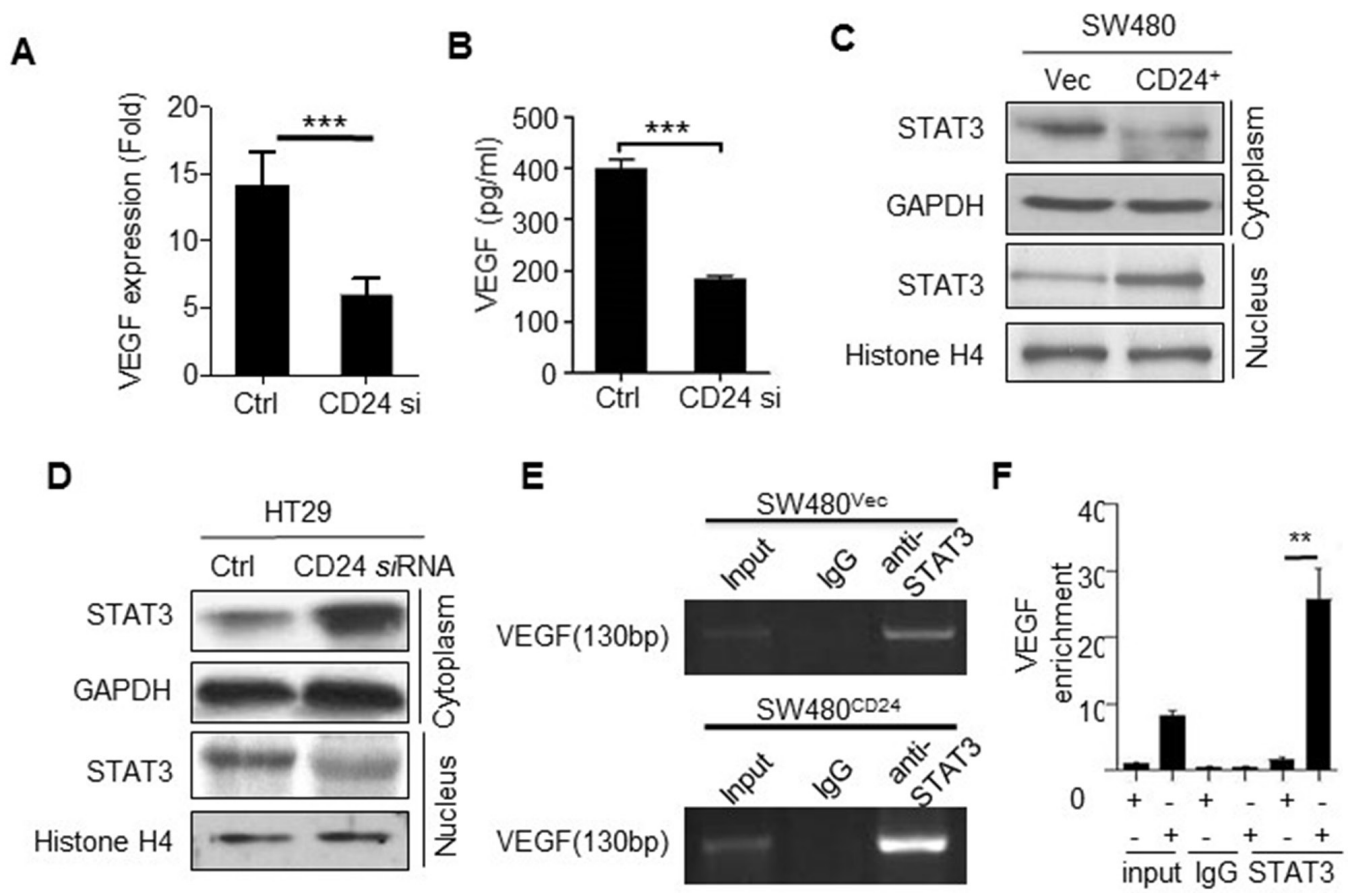
CD24- affected VEGF expression depending on the STAT3-mediated transcription **A, B.** VEGF mRNA and protein concentrations were analyzed by qPCR and ELISA in CM obtained from HT29 cells with the treatment of CD24 siRNA (CD24si) or control siRNA (Ctrl). ***, *p*<0.001, **C.** SW480 cells transfected with pcDNA4-CD24-Myc plasmid or control plasmid for 48h; **D.** HT29 cells were transfected with CD24 siRNA (CD24si) or control siRNA (Ctrl) for 48h. STAT3 expression in cytoplasm and nucleus were detected by Western blot. GAPDH and Histone H4 were used as internal control. **E, F.** CD24 promoted STAT3 to bind to the VEGF promoter. STAT3-DNA binding activity was determined by ChIP assay in SW480^Vec^ and SW480^CD24^ cells. Immunoprecipitation was conducted with anti-STAT3 antibody followed by using PCR oligonucleotide primers that yielded a 130bp band spanning STAT3 binding site in VEGF promoter. The PCR products were run on a gel for direct visualization (left) and qPCR was performed for quantitation (Right). Input lane represents 0.02% of total chromatin used in ChIP assays. **, *p*<0.01, SW480^CD24^ *vs* SW480^Vec^. CM: Conditional Medium.

VEGF constitutes one of the major proteins with pro-angiogenic activity. To elucidate the underlying mechanism of CD24-induced angiogenesis, we evaluated the effects of CD24 on VEGF secretion. Our results showed that VEGF mRNA decreased after CD24 siRNA treatment in HT29 cells (Figure 2A). Likewise, the secretion of VEGF in the supernatant was less than the control group (Figure 2B). On the contrary, both VEGF mRNA and protein were dramatically increased after SW480 cells were treated with transfecting CD24 overexpression plasmid and control in SW480 (data not shown). This data suggested that CD24 could regulate the expression of VEGF.

STAT3, a transcriptional factor, could modulate VEGF transcription and expression by binding to VEGF promoter region after its nuclear translocation [16]. As shown in Figure 2C, 2D, CD24 induced STAT3 translocation from cytoplasm to nucleus. To further verify whether the activity of STAT3 binding to the promoter of VEGF was affected by CD24, Chip assay was employed to detect the activity of STAT3 binding to the promoter of VEGF in the presence of silencing CD24 expression. The data showed that STAT3 was necessary for CD24-mediated VEGF expression. Moreover, luciferase assay was employed to verify the ability of STAT3 directly binding to the promoter of VEGF (Figure 2E, 2F). Our results demonstrated that phosphorylation of STAT3 mediated the CD24/VEGF signaling pathway. But as we known, CD24 was localized in the cell membrane, and no direct interaction with STAT3. So it is obscure how CD24 regulates of STAT3 translocation and transfers signals.

### Hsp90 is the predicated candidate protein interacted with CD24 in colorectal cancer cells

**Figure 3:**
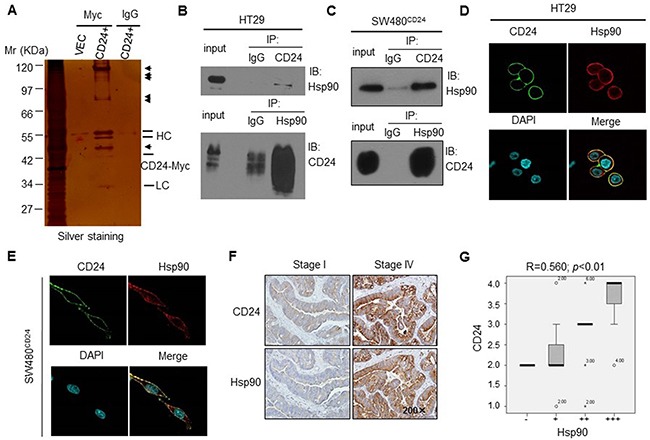
Hsp90 was one of the candidate protein interacting with CD24 in human CRC cells **A.** Identification of CD24-interacting proteins by Co-IP in HT29 cells. Silver staining of SDS-PAGE gel was shown. Arrows indicated the positions of potential CD24-interacting proteins. HC: Heavy chain; LC: Light chain; CD24+: pcDNA4-CD24-Myc plasmid; VEC: pcDNA4 Myc-His B vector. Confirmation of CD24-Hsp90 interaction in HT29 **B.** and SW480^CD24^ **C.** cells by Co-IP. The IP blot was probed with indicated antibodies. Input was 1% of the extract from untreated cells. Confocal microcopy showed that Hsp90 co-localized with CD24 in the HT29 **D.** and SW480^CD24^ **E.** cell. CD24 (Green) localized on the cell membrane; Hsp90 (Red) was distributed both on the membrane and cell plasma; DAPI was used to stain the nucleus (Light blue). **F.** The representative pictures of IHC staining for CD24 and Hsp90 in human CRC tissues with different stages (left: stage I; right: stage IV), original magnification: 200x. CD24 (upper) and Hsp90 (bottom) showed weak expression in primary cancer tissues (stage I) and strong expression in metastatic tissues (stage IV); **G.** CD24 and Hsp90 positive staining were quantified and the correlation was analyzed using Spearman correlation method, correlation coefficient R=0.560, p<0.01.

CD24 has no definite mechanism for signal transduction in tumor progression. To investigate how CD24 regulated the phosphorylation of STAT3 translocation from cytoplasm to nuclei, we used a mass spectrometry-based approach to identify CD24 protein interaction partners in HT29 cells. Since the antibody against CD24 (ML5) we used was not as sensitive as SWA11 (provided by Peter Altevogt), to maximize the pull down efficiency, we overexpressed CD24 with pcDNA4-CD24-myc plasmid in HT29 cells. After electrophoretic separation of the immunoprecipitated proteins, silver staining showed almost the same result that the presence of protein bands that specifically associated with CD24 (Figure 3A). Among the most apparent protein that have identified which were involved in STAT3 activation and VEGF expression or angiogenesis [23, 24]. Hsp90 was a special protein coimmunoprecipitated with CD24. We also detected the other screened proteins, such as Grp75 and Hspa8/Hsc71 by coimmunoprecipitated with CD24, and found that these two proteins were also interacted with CD24, but no evidences suggested that both two proteins were involved in tumor angiogenesis (Supplementary Figure S2). Thus we focused on Hsp90 and investigated whether it played as a transistor between CD24 and downstream signals. To confirm the interaction of Hsp90 and CD24, endogenous CD24 was pulled down and detected with anti-Hsp90 antibody by western blot in HT29 cells (Figure 3B, upper); Hsp90 was also pulled down and detected with anti-CD24 antibody (Figure 3B, bottom). In addition, the similar results was repeated with overexpressed CD24 in SW480 (Figure 3C). Our results identified the predicated interaction between Hsp90 and CD24 by mass spectrometry analysis. Furthermore, we also observed the cellular co-localization CD24 and Hsp90 by immunofluorescence analysis. As shown in (Figure 3D, 3E), Hsp90 co-localized with CD24 in CRC cell membrane. To confirm the correlation between CD24 and Hsp90 in human colorectal cancer patients tissue, IHC staining was performed in serial sections of human CRC tissues. Semi-quantitatively, scoring of the two proteins showed that the expression of both proteins in cancerous tissues was significantly higher than that of adjacent normal tissues (*p*<0.05, Figure 3F). Spearman correlation analysis showed a positive correlation between CD24 and Hsp90 expression (correlation coefficient R=0.560, *p*<0.01, Figure 3G). Our results demonstrated that CD24 was directly interacted to Hsp90.

### CD24 recruited Hsp90α at lipid raft

**Figure 4:**
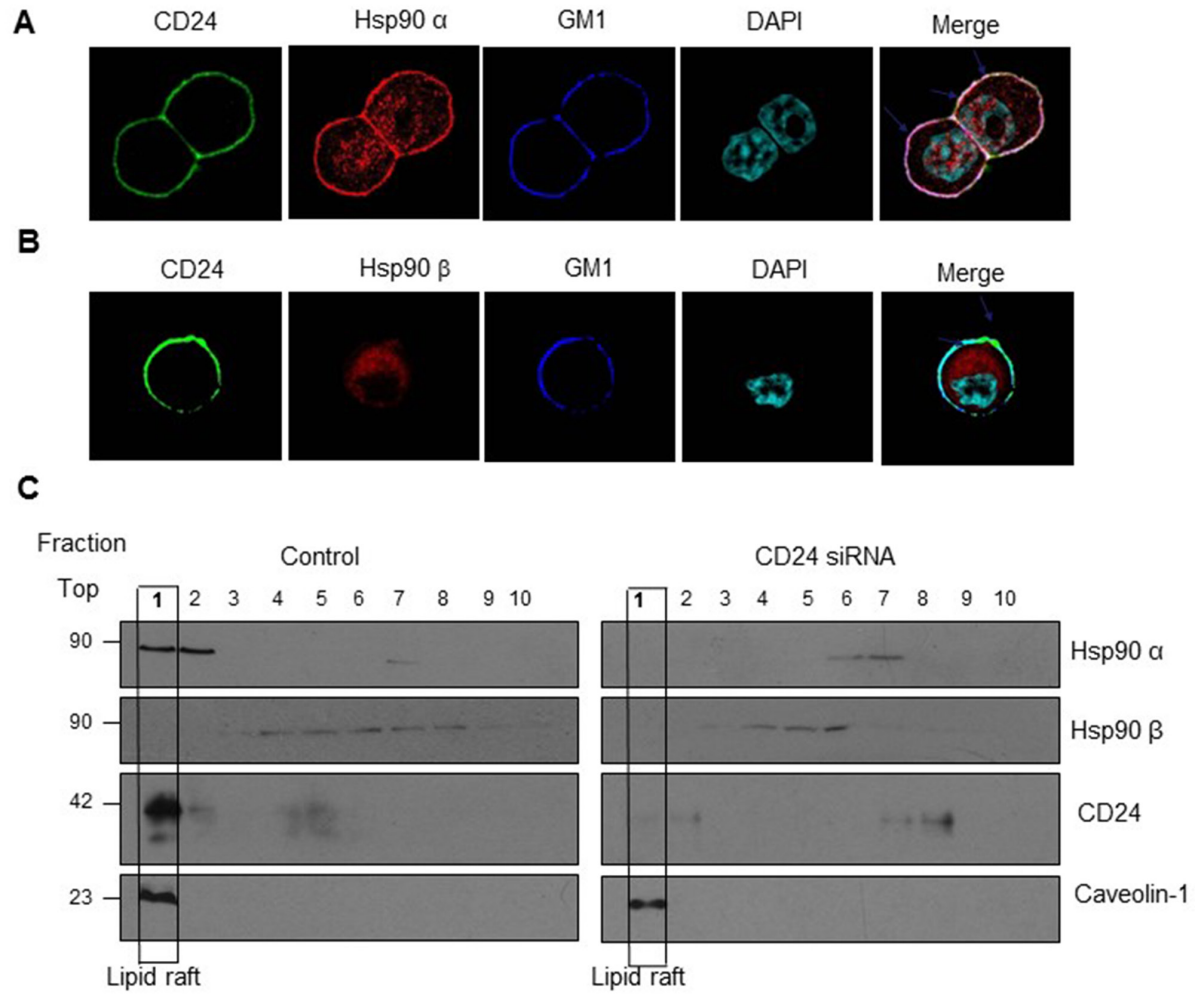
Co-localization of Hsp90α and CD24 in lipid raft **A.** Confocal microcopy showed that Hsp90α was co-localized with CD24 and GM1 (lipid raft marker). CD24 (Green) as well as GM1 (Blue) localize on the cell membrane; Hsp90α (Red) is distributed both on the membrane and in the cytoplasm while Hsp90β (Red) only in the cytoplasm; DAPI was used to stain the nucleus (Light blue). Merged confocal image showed co-localization of Hsp90α, CD24 and GMI by the white staining on the cell membrane (arrows). Scale bar: 100μm. **B.** Lipid raft localization of Hsp90α and CD24 in HT29 cells (lane1, left) and treatment with CD24 siRNA reduces the association of Hsp90α with CD24 on rafts (lane1, right). **C.** HT29 cells treated with control or CD24 siRNA were lysed to obtain the detergent-soluble (bottom) and detergent-resistant fractions as described in “Materials and Methods”. Aliquots of each fraction were analyzed by Western blot to detect the expression of Hsp90α, Hsp90β, CD24 and lipid raft marker Caveolin-1. One representative profile of three experiments was shown.

GPI-linked proteins are often associated with cholesterol-rich lipid raft micro-domains in the plasma membrane. CD24 belongs to the GPI-linked protein, and has been reported to recruit β1 intergrin, Met and interact c-Src to lipid raft domains, which activated the downstream signaling pathway [20, 25, 26]. In order to further explore the underlying mechanism of Hsp90 and CD24 interactions, we addressed our target to lipid raft as it is thought to serve as platforms that aggregate specific proteins, including CD24, to facilitate cell signaling. We immuno-stained Hsp90α or Hsp90β, CD24 and the lipid raft marker (GM1) in HT29 cells and found that Hsp90α partly co-localized with CD24 in lipid raft, but not Hsp90β (Figure 4A, 4B). We also observed the co-localization of Hsp90α and CD24 in lipid raft fractions of HT29 cells by Western blot (Figure 4C, lane1, left). Moreover, Silencing CD24 with siRNA dramatically reduced the expression of Hsp90α and CD24 in lipid raft fractions (Figure 4C, lane1, right). Collectively, CD24 could recruit Hsp90α at lipid raft, but not Hsp90β.

### Hsp90 maintained the stabilization of CD24

Hsp90 is known to stabilized and activate multiple proteins and blocking the association of Hsp90 with its substrates by disrupting its ATPase function, which leads to the degradation of these client proteins [27, 28]. To further explore the role of Hsp90 in CD24 protein stabilization, we then detected the effects of 17-AAG, the special inhibitor for Hsp90, on CD24 protein expression [29].

**Figure 5:**
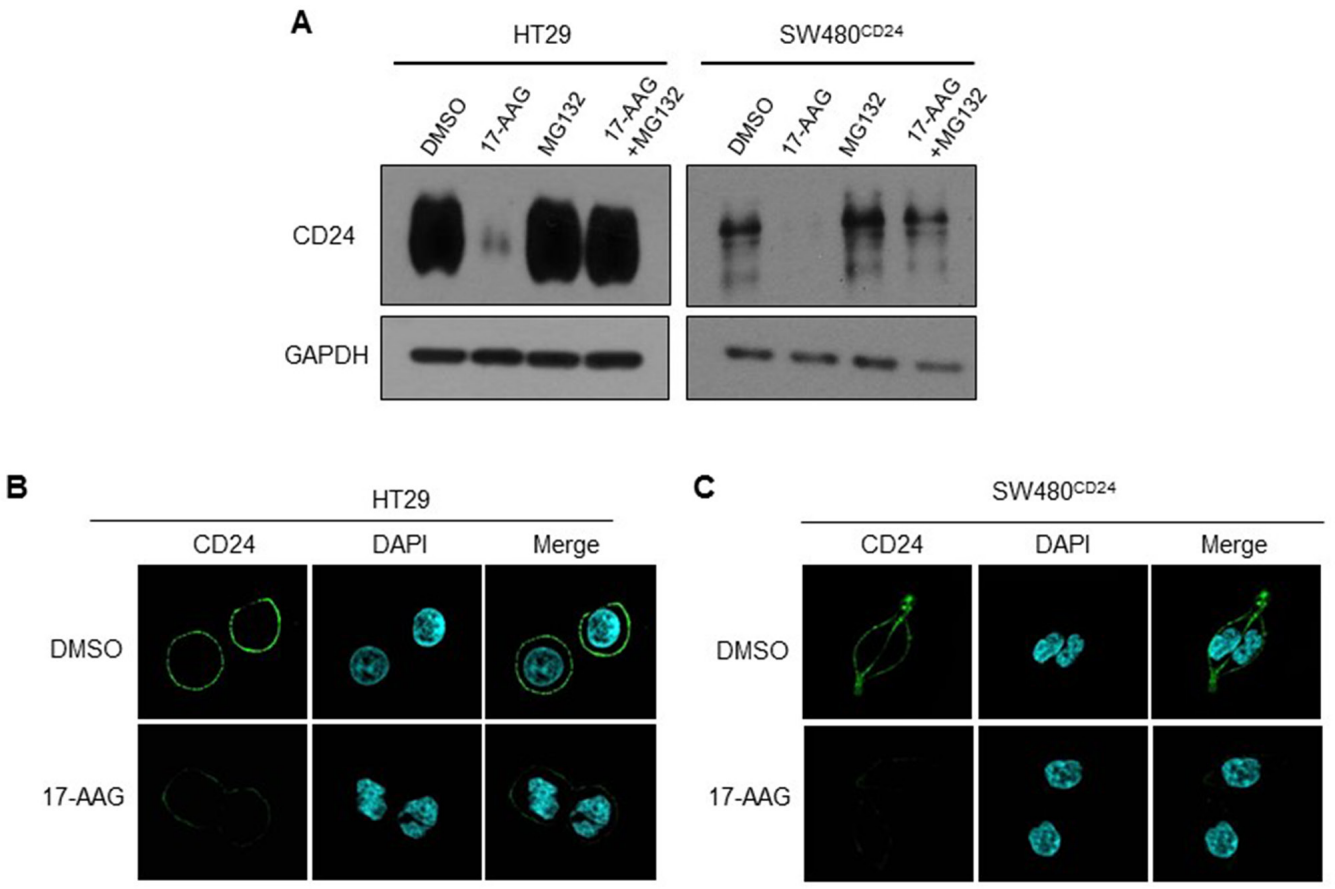
Hsp90 stabilized CD24 in CRC cells **A.** HT 29 and SW480^CD24^ cells were treated with MG132 (2μM) and/or 17-AAG (1μM) for 24h. Western blot was performed to detect the expression of CD24. GAPDH was used as an internal control. A representative photograph of three experiments was shown. Fluorescent confocal micrographs of decreased membranous CD24 staining in 17-AAG-treated HT29 **B.** and SW480^CD24^ cells **C.** HT29 and SW480^CD24^ cells treated with or without 17-AAG (1μM for 24h) were fixed, permeabilized and stained with DAPI (blue) and CD24 (green).

The results of western blot and immunofluorescence staining showed that CD24 expression reduced after 17-AAG treatments in HT29 cells and SW480^CD24^ cells (Figure 5A, 5B and 5C). To address if down-regulation of CD24 induced by 17-AAG was due to the degradation mediated by proteasome, HT29 cells and SW480^CD24^ cells were pretreated with proteasome inhibitor MG-132 and further cultured with 17-AAG in CRC cells (Figure 5A: line 3, 4;). Our results showed that MG-132 reversed the reduction of CD24 expression induced by 17-AAG, indicating that Hsp90 protected CD24 from degradation and maintained its stability.

### 17-AAG attenuated the CD24-dependent VEGF and angiogenesis *in vitro* and *in vivo*

**Figure 6:**
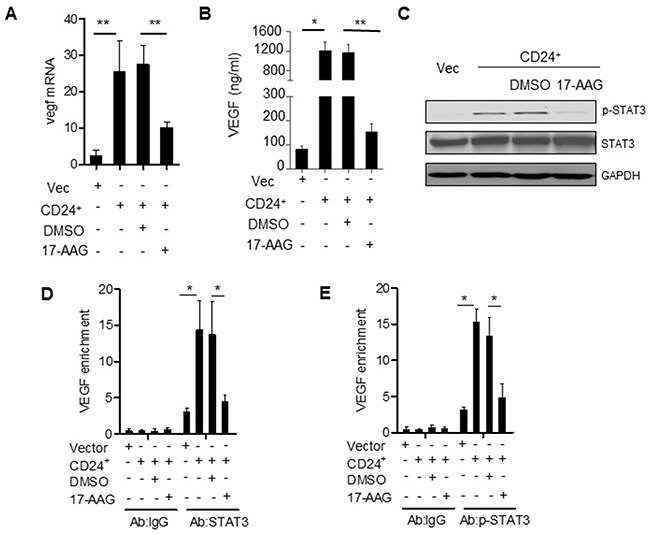
Hsp90 is the mediator of CD24--VEGF signaling pathway VEGF mRNA **A.** and protein concentrations **B.** were analyzed by qPCR and ELISA in CM obtained from SW480 cells transfected with pcDNA4-CD24-Myc plasmid or control plasmid as indicated 24h later. 1μM 17-AAG or 0.1% DMSO was added 1h before the transfection. *, *p*<0.05, SW480^CD24^ *vs* SW480^Vec^; **, *p*<0.05, SW480^CD24^+DMSO *vs* SW480^CD24^+17-AAG. **C.** STAT3 and p-STAT3 were detected by Western blot as indicated. **D, E.** CD24 promoted STAT3 and p-STAT3 to bind to the VEGF promoter. STAT3-DNA binding activity was determined by ChIP assay in SW480^Vec^ and SW480^CD24^ cells, 1μM 17-AAG or 0.1% DMSO was added 1h before the transfection. Immunoprecipitation was conducted with STAT3 or p-STAT3 antibody followed by qPCR using oligonucleotide primers Input lane represents 0.02% of total chromatin used in ChIP assays. *, *p*<0.05, SW480^CD24^ *vs* SW480^Vec^.

**Figure 7:**
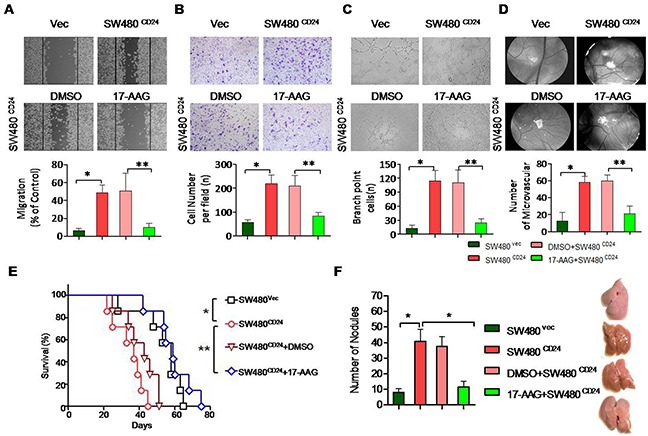
Hsp90 contributed to the CD24-induced angiogenesis *in vitro* and *in vivo* CD24-induced HUVECs’ migration **A.** invasion **B.** and tubule formation **C.** was abolished by 17-AAG. CM was obtained from SW480 cells transfected with pcDNA4-CD24-Myc plasmid or control plasmid as indicated 24h later. 1μM 17-AAG or 0.1%DMSO was added 1h before the transfection. **D.** Angiogenic responses induced by gelatin sponges loaded with CM obtained as indicated *in vivo* CAM model. Original magnification: 40x. Vessel counts under a stereomicroscope at the sponge-CAM boundary were indicated beside the image. Photographs were representatives of three independent experiments. Error bar represent mean±SEM. *, *p*<0.05, SW480^CD24^ *vs* SW480^Vec^; **, *p*<0.05, SW480^CD24^+DMSO *vs* SW480^CD24^+17-AAG. CD24: pcDNA4-CD24-Myc plasmid; Vec: pcDNA4 Myc-His vector. **E** and **F.** Experimental liver metastases were generated by intrasplenic injection of 2×10^4^/100μl cancer cells. Each group included six mice. Mice survival was analyzed with long-rank test.

The previous document demonstrated that inhibition of Hsp90 could attenuated the levels of STAT3 and phospho-ERK in various tumors [30, 31]. As shown previously, the conditioned medium (CM) obtained from SW480 cells transfected with CD24 overexpression plasmid stimulated HUVECs. The treatment of 17-AAG could abolish VEGF expression (Figure 6A, 6B) and inhibit the STAT3 translocation and phosphorylation (Figure 6C). As a result, 17-AAG decreased the activity of STAT3-binding to the promoter of VEGF, which was confirmed by Chip assay (Figure 6D). In addition, the HUVECs migration increased after the overexpression of CD24 (*p*<0.05), but was inhibited with 17-AAG-treatment (*p*<0.05). The ability of the HUVECs invasion and tubule formation decreased in the presence of 17-AAG (Figure 7A, 7B, 7C and 7D). The number of newly formed blood vessels was also decreased significantly in 17-AAG-treated disk by CAM assay (*p*<0.05). *In vivo*, the mouse liver metastasis model was employed to evaluate the metastatic ability of the colorectal cancer cells [32]. Our data showed that metastatic nodules increased in CD24 overexpressed cells, but decreased after the treatment of 17-AAG; and survival time obviously prolonged in the presence of 17-AAG (Figure 7E,F). Our data suggested that Hsp90 contributed to the CD24-induced angiogenesis *in vitro* and *in vivo*.

## DISCUSSION

Recent studies showed that CD24 was involved in tumor progression including angiogenesis. However, the underlying mechanism is still unknown. In this study, we found that CD24 induced HUVEC migration, invasion, and tubule formation *in vitro* and supposed whether CD24 could promote CRC progression through the regulation of angiogenesis. To elucidate the mechanism, we assessed CD24-interacted protein using high throughout mass spectrometry and immune-precipitation. Among these potential interacting proteins, Hsp90 was the most important one. As a molecular chaperone, Hsp90 interacts with multiple co-chaperones to assure the maintenance of protein quality in the cell by regulating the balance between folding and degradation of proteins. Many of its client proteins are involved in angiogenesis including EGFR, Met and MMPs [26, 27]. So, we hypothesized that Hsp90 was involved in CD24-induced angiogenesis, and aimed to investigate the role and underlying mechanism CD24 in CRC angiogenesis and the underlying mechanism.

Accumulated evidences demonstrated that CD24 belonged to the glycosylphosphatidylinositol-anchored membrane protein, and the targeting CD24 therapeutic strategies have been positively verified in many human carcinomas by inhibiting tumor proliferation and metastasis [33]. But in previous studies, tumor angiogenesis was rarely mentioned. Angiogenesis is an important step for tumor carcinogenesis and metastasis. Our results showed that CD24 expression was closely related to MVD, especially in liver metastasis tissues of human CRC patients. These datas suggested that CD24 possiblely promoted the CRC metastasis by regulating the expression of VEGF, which was responsible for tumor MVD [34]. We further confirmed that alerted CD24 expression could affect CRC cells migration, invasion and tubule formatting. It remained unclear whether CD24 could regulate the expression of VEGF in CRC. Previous studies revealed that CD24 mediated gastric carcinogenesis and promoted cancer cell progression *via* STAT3 activity, and regarded various CD24-mediated genes as STAT3 target genes [25, 35]. STAT3 was involved in many tumor development and progression, and also played an important role in cancer stem cell [36, 37]. Moreover, STAT3 was regarded as the key transcription factor to the promoter of VEGF in normal and tumor cells. Our data also verified that CD24 controlled STAT3 activity and translocation in CRC, and STAT3 activated the VEGF expression *via* binding to its promoter.

However, no evidence demonstrated how CD24 regulate the STAT3 activity. Next, we performed the mass spectrometry to investigate which proteins assisted CD24 to regulate the STAT3 activity. Co-IP and immunofluorescence staining results provided evidences for CD24 and Hsp90 interaction. Hsp90 was a chaperone protein, which was reported to play a pivotal role in protein maturation, degradation and the regulation of biological function including tumor migration, invasion and angiogenesis, was highly enriched in mass spectrometry. CD24 is mostly composite of glycosylation and protein- polysaccharide interactions largely depend on cations, which promotes the Hsp90 combination with CD24. This interaction was also verified by Y. Liu *et al* in immune cells [38]. On the other hand, the previous research supported that CD24 could act as a “gate-keeper” for lipid raft domain, which recruited the other proteins and regulated their activity, such as β1 intergrin, Met and other proteins. Our data also suggested that CD24 could recruit Hsp90a, but not Hsp90β, at lipid rafts [26]. HGMB1 was regarded as an interaction protein of CD24, but we could not find the interaction between HGMB1 and CD24 in current reports, which may be due to the use of a different cell type. In addition, Hsp90 could directly activate STAT3 via binding to its N-terminal and in turn regulated VEGF expression [39, 40].

Hsp90 was reported to maintain the “client protein” stability and functionally modulate signal transduction. Blocking Hsp90 activity not only promoted the “client protein” disability and degradation, but also inhibited the signal transduction. 17-AAG, a kind of Hsp90 inhibitor which binding to the ATP site of Hsp90 and disrupting its association with client proteins, was regarded as the phase II trial anti-tumor drug in various of tumors [41, 42]. In current research, we found that 17-AAG significantly inhibited CD24-induced HUVECs migration, invasion and tubule formation in *vitro*; 17-AAG also inhibited angiogenesis *in vivo*. These data suggested that Hsp90 was involved in CD24-induced CRC angiogenesis.

To explore the mechanism, we detected the downstream signaling pathway. In our previous project, we have identified MAPKs pathway which was involved in CD24-induced CRC tumorigenesis. Another target is STAT3, which is a point of convergence for many tyrosine kinase signal pathways and is constitutively activated at high frequency in a wide range of cancers. More important, STAT3 can bind to the promoter region (position-848) of VEGF and regulate the transcription and expression of VEGF directly, which indicated STAT3/VEGF pathway act as an important role in tumor angiogenesis. STAT3 has two major phosphorylation sites, Tyr705 and Ser727. We found only phospho-STAT3 (Tyr705) expression increased with CD24 overexpression, but not Ser727, and this effect was abrogated by 17-AAG-treatment. Our data also showed a cytosolic decrease and a nuclear accumulation of STAT3 when CD24 was overexpressed. Moreover, Chip assay using anti-STAT3 antibody confirmed the STAT3/VEGF DNA binding ability in SW480^CD24^ cells. Our results indicated that CD24 regulated VEGF expression *via* STAT3-mediated transcription of VEGF (Supplementary Figure S3).

Collectively, we concluded that CD24 was involved in colorectal cancer angiogenesis in Hsp90-dependent manner. STAT3-mediated transcription of VEGF was involved in this process. Our studies provided a new insight of CD24 as an anti-angiogenesis agent during CRC progression and migration.

## MATERIALS AND METHODS

### Reagents and antibodies

MG132(Z-Leu-Leu-Leu-al) (Sigma) and 17-(Allylamino) - 17-demethoxygeldanamycin (17-AAG) (Sigma) were prepared in 0.1% DMSO (Sigma St. Louis, MO) or PBS. Mouse anti-CD24 monoclonal antibody (SWA11) was gifted from Peter Altevogt (German Cancer Research Center, Germany). Mouse anti-myc antibody was purchased from Roche Applied Science (Basel, Switzerland). Mouse anti-Hsp90 and Rabbit anti-Hsp90 antibodies were obtained from R&D Systems (Abingdon, UK). Goat anti-mouse IgG and Goat anti-rabbit IgG were purchased from Jackson-Immunoresearch (Suffolk, UK). Anti-mouse IgG (whole molecule) FITC-conjugate and anti-rabbit IgG TR conjugate were from Sigma (St. Louis, MO). The following antibodies were used: Normal mouse IgG, mouse anti CD24 (ML5), GAPDH, phospho-ERK1/2(Thr202/Tyr204), ERK1/2, p38MAPK, phospho-p38MAPK (Thr180/Tyr186), phospho-STAT3 (Tyr705), phospho-STAT3 (Ser727), STAT3 and Histone H4 antibodies (Cell signaling Technology, MA). Matrigel was purchased from BD Biosciences (NY, USA). Fertilized chicken eggs were purchased from South China Agricultural University (Guangzhou, China).

### Plasmids construction, siRNA and transfection

The full-length human CD24 was amplified by PCR as previously described [5]. The primers for CD24 were: Forward, 5′-gttgttGGATCCATGGGCAGAGCAATGGT-3′; Reverse, 5′-gttgttCTCGAGcgAGAGTAGAGATGCAGAAGAGAG-3′. The pcDNA4-CD24-myc plasmid was constructed by inserting this PCR product into the pcDNA4 Myc-His B vector (Invitrogen, Carlsbad, CA) and verified by sequencing. The sequences of CD24 *si*RNA and a control *si*RNA were as follows: 5′-UCUCUCUUCUGCAUCUUUAdTdT-3′ and 5′-UUCU CCGAACGUGUCACGUTT-3′ respectively. Transfections of plasmids or siRNA were all done using the lipofectamine 2000™ reagent (Invitrogen, Carlsbad, CA) according to the manufacturer's instructions. Stably transfected clones were selected in cultured medium containing 800μg/mL G418 and analyzed for CD24 expression levels by Western blot. GAPDH was used as the internal control.

### Cell line and cell culture

HT29 and SW480 cell lines were obtained from American Type Culture Collection (ATCC, Manassas, VA). SW480^CD24^ and SW480^Vec^ cells were established as described in our previous study [19]. HT29, SW480, SW480^CD24^ and SW480^Vec^ cells were cultured in RPMI1640 supplemented with 10% fetal bovine serum (FBS), 100 units/ml penicillin, and 100μg/ml streptomycin. Human umbilical vein endothelial cells (HUVECs) were obtained from Cell Bank of Shanghai Institutes for Biological Sciences (Shanghai, China) and cultured in ECGM medium supplemented with 20% FBS, 1% BBE/ heparin mixture, 1% antibiotics, and 0.5% fungizone. All cells were maintained at 37°C in a water-saturated 5% CO_2_ incubator. Hsp90 inhibition was accomplished by treating cell monolayers with 17-AAG (1μM) for 24h. MG-132 (2μM) was utilized to inhibit proteasome activity.

### Mass spectrometry analysis

Protein lysates from HT29 cells, transfected with pcDNA4 or pcDNA4-CD24-myc plasmids, were prepared for immune-precipitation as described below. After separation by 12.5% SDS-PAGE gel, bands of interest identified by colloidal Coomassie Blue (Invitrogen, Carlsbad, CA) staining were excised manually from gel for mass spectrometry identification. The peptides were analyzed by liquid chromatography-tandem mass spectrometry (LC-MS/MS) with a MALDI-TOF/TOF UltraFlex II (Bruker Daltonics, Bremen, Germany) mass spectrometer. The target proteins were identified by comparison to the human MSDB (Mass Spectrometry protein sequence Database) databank.

### Co-immunoprecipitation (Co-IP) and western blot

Total protein lysates from SW480^CD24^, SW480^Vec^ cells or HT29 cells were obtained by incubation on ice for 30min in immunoprecipitation (IP/lysis) buffer (50mM HEPES, pH 7.5, 150mM NaCl, 5mM EDTA, 1% Triton X-100, 1mM PMSF, 2μg/mL Pepstatin A, and 1×cocktail protease inhibitors) followed by a centrifugation of 15000 g for 20min at 4°C. Some 25μl of protein G beads (Zymed Laboratories Inc.) were added to preclear the supernatant at 4°C for 1h. Afterward, the samples were incubated with 5~10μg of the indicated antibody or control mouse IgG (Pierce) for 1h and then with 30μl of protein G beads for 2h. After extensive washing, precipitates were subjected to Western blot for the detection of interacting proteins as described previously [19].

### Confocal microscopy

HT29 and SW480^CD24^ cells were plated on 12mm glass coverslips in culture dishes. 1μM 17-AAG was added after the adherence of cells. 24h later, cells were fixed with 4% paraformaldehyde in PBS for 15min at room temperature. The fixed cells were blocked in blocking buffer (2.5% BSA in PBS) and the un-permeabilized cells were incubated with primary anti-CD24 (SWA11) primary antibody and secondary fluorescent antibody to label CD24. Cells were visualized with an Olympus FV10i-W confocal microscope (Olympus, Inc., Japan).

For co-localization of CD24 and Hsp90, cells grown on coverlids were fixed with 4% paraformaldehyde for 20min at room temperature, and then permeabilized with 0.05% TritonX-100 in PBS. After blocking with 2.5% BSA for 1h at room temperature, cells were incubated with primary antibodies in blocking buffer containing 3% BSA for 1h, washed 3 times with PBS, and incubated with secondary fluorescent antibodies for 1h. The co-localization analysis was performed. The 488 and 543nm were used for detecting the expression of CD24 and Hsp90 respectively.

### Tissue specimen and immunohistochemistry (IHC)

Formalin-fixed, paraffin-embedded tissue samples from 81 primary CRC patients were randomly obtained and processed by routine clinical histopathological methods. The tumor stages were determined according to the TNM classification of the American Joint Committee on Cancer Criteria, and the World Health Organization (WHO) classification of tumors was used to determine the histological grade. The study was carried out in accordance with the institutional ethical guidelines and had been approved by the Medical Ethics Committee of Southern Medical University. The expression of CD24, CD31 and Hsp90 in colorectal cancer tissue was visualized by immunohistochemistry as previously described [19].

### Wound healing, migration and invasion assays

The supernatant medium, which was respectively derived from HT29 cells transfected with CD24 siRNA and control siRNA after 24h culture in conditions of RPMI1640 plus 10% FBS, was collected and filtrated twice with 0.22 μm sterile Millipore.

Two types of cell migration assays were done using HUVECs. For scratch assay, cells seeded in 6-well plates were scratched with 10μl pipet tip, and washed with 1×PBS for three times, further cultured for 24h with conditioned medium. Cells migrated toward the wound regions were imaged and counted.

The invasive and migratory potential of cells was evaluated using trans-well chamber with 8μm pores (BD bioscience, NY, USA). For migration assay, 2.0×10^5^ HUVECs re-suspended in serum-free medium were added to each upper chamber, which was pre-coated with 50μl Matrigel matrix (BD, NJ, USA). 500μl 10% conditioned medium was added into the matched lower chamber. After 24h incubation, non-invading cells were removed with cotton swabs, and the underside of the insert was stained with 4% formaldehyde and 0.1% crystal violet. Six random fields at 100× magnification for each chamber were counted using Olympus I×70 invert microscope (Olympus). Chambers were conducted in triplicate and repeated three times.

### Tubule formation assay

For tubule formation assay, 4.0×10^3^ cells were seeded into 96-well plates coated with Matrigel for 4h at 37°C. The tubule length was calculated in three random fields per well. Experiments were performed in triplicate and repeated three times.

### Chicken embryo CAM assay

According to previous method embryonic eggs were incubated in 38.5°C to 39°C with the relative humidity at 65% to 70%. Five days later, a diameter of 2 cm^2^ window was opened. The shell membrane was removed to explore the chick embryo chorioallantoic membrane (CAM). As the pellet, 6-mm-diameter medical gelation sponge sterilized filter disk, which absorbed the collected supernatant medium as described above, was put on the CAM. Only 1×PBS in the pellet was regarded the control group. The window was sealed with plastic wrap, and eggs were incubated again. Antibiotic (Ampicillin) was applied to prevent infection. Four days later, the CAM was observed under stereomicroscope and the neovascularization was measured, each experiment was triplicate.

### ELISA assay

Conditioned medium with transfecting CD24 overexpression plasmid in SW480 or CD24 siRNA in HT29 was collected as indicated. The supernatant was diluted to 1:1. The VEGF concentration was measured by an ELISA kit (Cusabio, China) with purified coating and biotinylated detection VEGF in cell supernatant following the manufacturer's instruction.

### Preparation of cytoplasmic and nuclear extract

Cells were re-suspended in 400μl buffer A (containing 10mM Hepes, pH 7.9, 1.5 mM MgCl_2_, 10mM KCl, 0.5mM DTT, 0.5mM phenylmethylsulfonyl fluoride, 1μg/ml leupeptin, 1μg/ml aprotinin, and 1μg/ml pepstatin A), lysed with 12.5μl of 10% Nonidet P-40, and centrifuged at 12,000 g for 10min at 4°C. The supernatant was collected and used as the cytoplasmic extracts. The nuclei pellet was re-suspended in 40μl of buffer B (20mMHepes, pH7.9, containing 1.5mM MgCl_2_, 450mM NaCl, 25% glycerol, 0.2mM EDTA, 0.5mM DTT, 0.5mM phenylmethylsulfonyl fluoride, 1μg/ml leupeptin, 1μg/ml aprotinin, 1μg/ml pepstatin A) and agitated for 60min at 4°C, and the nuclear debris was spun down at 20,000 g for 15min. The supernatant (nuclear extract) was collected and stored at 80°C until ready for analysis. Protein concentrations were determined with BCA protein assay kit.

### Chromatin immunoprecipitation (ChIP) assays

According to the protocol of CHIP assay kit (Upstate Cell Signaling Solutions, Lake Placid, NY), a total of 3×10^7^ asynchronously growing SW480^Vec^ and SW480^CD24^ cells were cross-linked by 1% formaldehyde respectively. After sonication, the chromatin solution was diluted 10-fold with ChIP Dilution Buffer and precleared with protein A agarose /salmon sperm DNA. The precleared chromatin solution was divided and incubated with either an anti-STAT3 polyclonal antibody or normal rabbit IgG. Protein A agarose /salmon sperm DNA was added to each fraction and rotated at 4°C. Then the antibody/protein/DNA complex were washed and eluted off the beads using elution buffer. Crosslinking was reversed by heating at 65°C. DNA was purified and subjected to routine PCR and qPCR with primers specific for a 130-bp region (−913 to −783) spanning the STAT3-binding site (−848) in the VEGF promoter [23]. The sequences of the PCR primers used are as follows: VEGF forward (+): 5′-CTGGCCTGCAGACATCAAAGTGAG-3′ and VEGF reverse (−): 5′-CTTCCCGTTCTCAGCTCCACAAAC-3′.

### Lipid raft isolation

As described in previous studies [20, 21], 5×10^6^ cells were detached from tissue culture plastic surface treated with 0.1M PBS, 5 mM EDTA and lysed in ice-cold lysis buffer [20 mmol/L Tris/HCl, pH 8.0, containing 50 mmol/L b-octylglycopyranoside (BOG) or 1% Triton X-100, 10 mmol/L NaF, 10 mmol/L orthovanadate, 1 mmol/L PMSF,1μg/ml of each leupeptin, aprotenin, and pepstatin] for 30 min on ice. The lysates were mixed with an equal volume of 85% sucrose (w/v in TBS), and step gradient was prepared by overlaying with 35% sucrose (w/v in TBS) followed by a final layer of 5% sucrose. The gradient was centrifuged for 20h at 200,000g. Fractions of 500 μl were collected from the top of the gradient, preciptitated with a tenfold volume of acetone and then washed with a fivefold volume of 50% acetone in H_2_O. Samples were dried and mixed with non-reducing SDS-sample buffer for Western blot.

### Liver metastasis model

This study was performed in accordance with the institutional guidelines and approved by the Animal Experimentation Committee of Southern Medical University. Cells was harvested and suspended in 0.1M PBS at a concentration of 2×10^5^/ml. Experimental liver metastases was generated by intrasplenic injection of 2×10^4^/100 μl cancer cells and splenectomy. The mice were sacrificed 6 weeks later, and liver metastaseswas enumerated [22].

### Statistical analysis

Results *in vitro* and *in vivo* were statistically evaluated using the standard two-tailed student's *t* test or one-way ANOVO. P-value less than 0.05 or 0.01 or 0.001 were considered significant.

## SUPPLEMENTARY FIGURES


