# Supplementary material for: CD24 promoted cancer cell angiogenesis via Hsp90-mediated STAT3/VEGF signaling pathway in colorectal cancer
Source: Oncotarget. 2016 Aug 1;7(34):55663–76. doi: 10.18632/oncotarget.10971 (PMC5342444; doi:10.18632/oncotarget.10971)
Supplement: Supplementary file 1 [file oncotarget-07-55663-s001.pdf]

CD24 promoted cancer cell angiogenesis via Hsp90-mediated STAT3/VEGF signaling pathway in colorectal cancer

SUPPLEMENTARY FIGURES

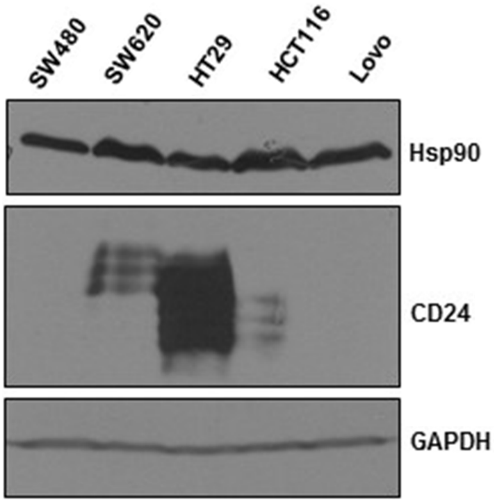

Supplementary Figure S1: The expression of CD24 and Hsp90 in different colorectal cancer cell lines. GAPDH was used as a negative control.

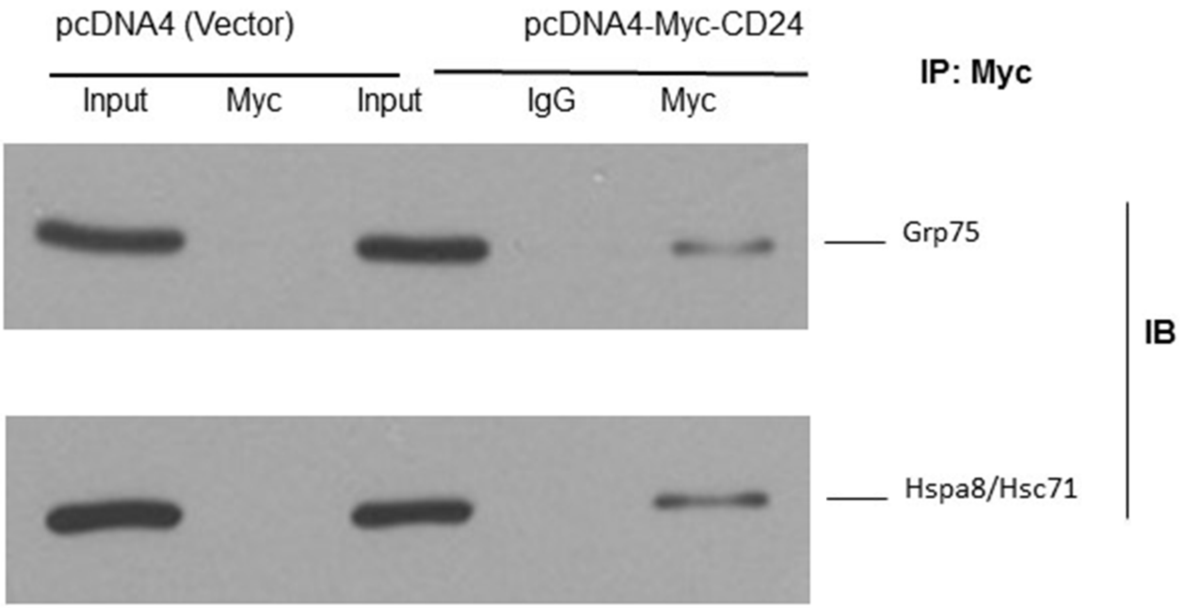

Supplementary Figure S2: Grp75 and Hspa8/Hsc71 by coimmunoprecipitated with CD24.

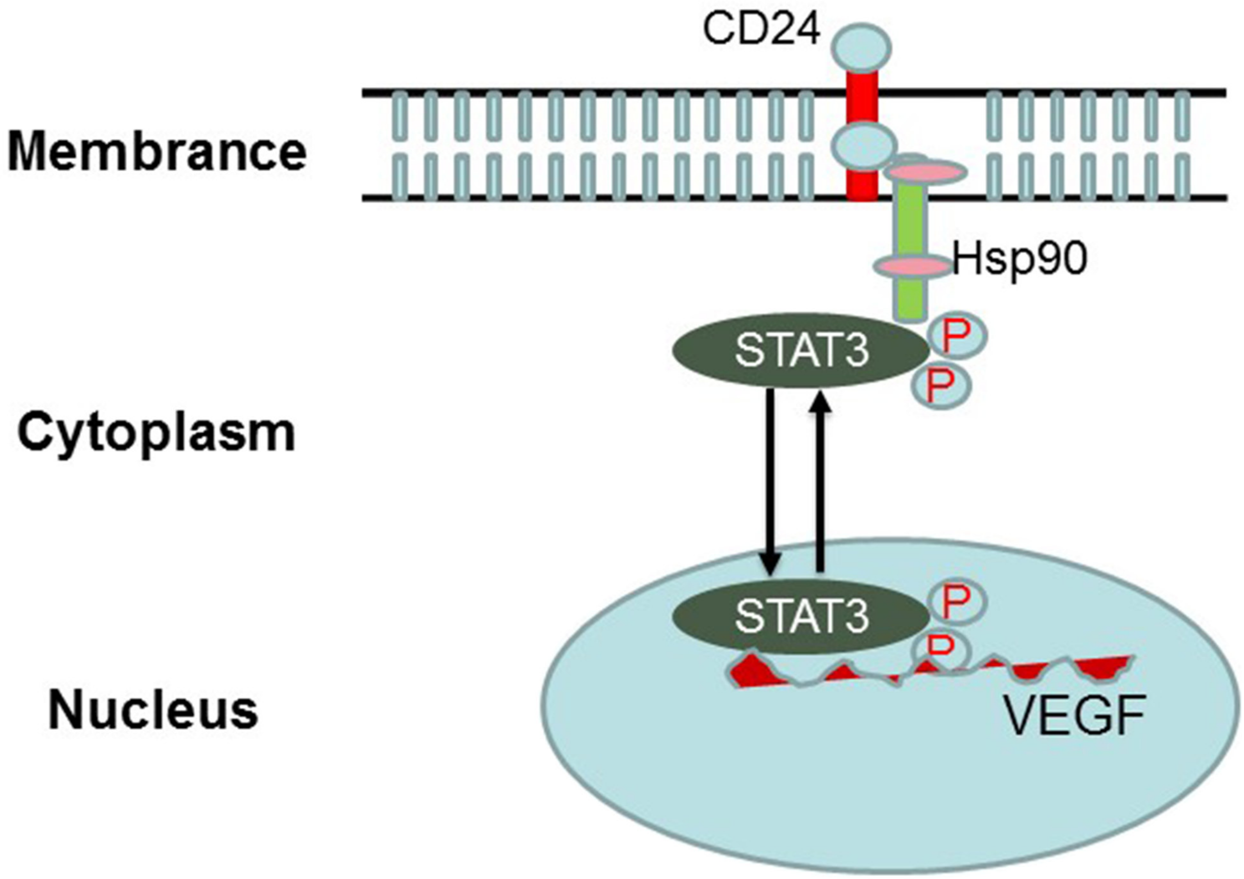

Supplementary Figure S3: A diagram summarizing the relationship of CD24-HSP90-STAT3.
